# Supplementary material for: Targeting Peptide, Fluorescent Reagent Modified Magnetic Liposomes Coated with Rapamycin Target Early Atherosclerotic Plaque and Therapy
Source: Pharmaceutics. 2022 May 18;14(5):1083. doi: 10.3390/pharmaceutics14051083 (PMC9146689; doi:10.3390/pharmaceutics14051083)
Supplement: Supplementary file 1 [file pharmaceutics-14-01083-s001.zip › pharmaceutics-1691270-supplementary.pdf]

Supplementary Material

1 Supplementary Figures

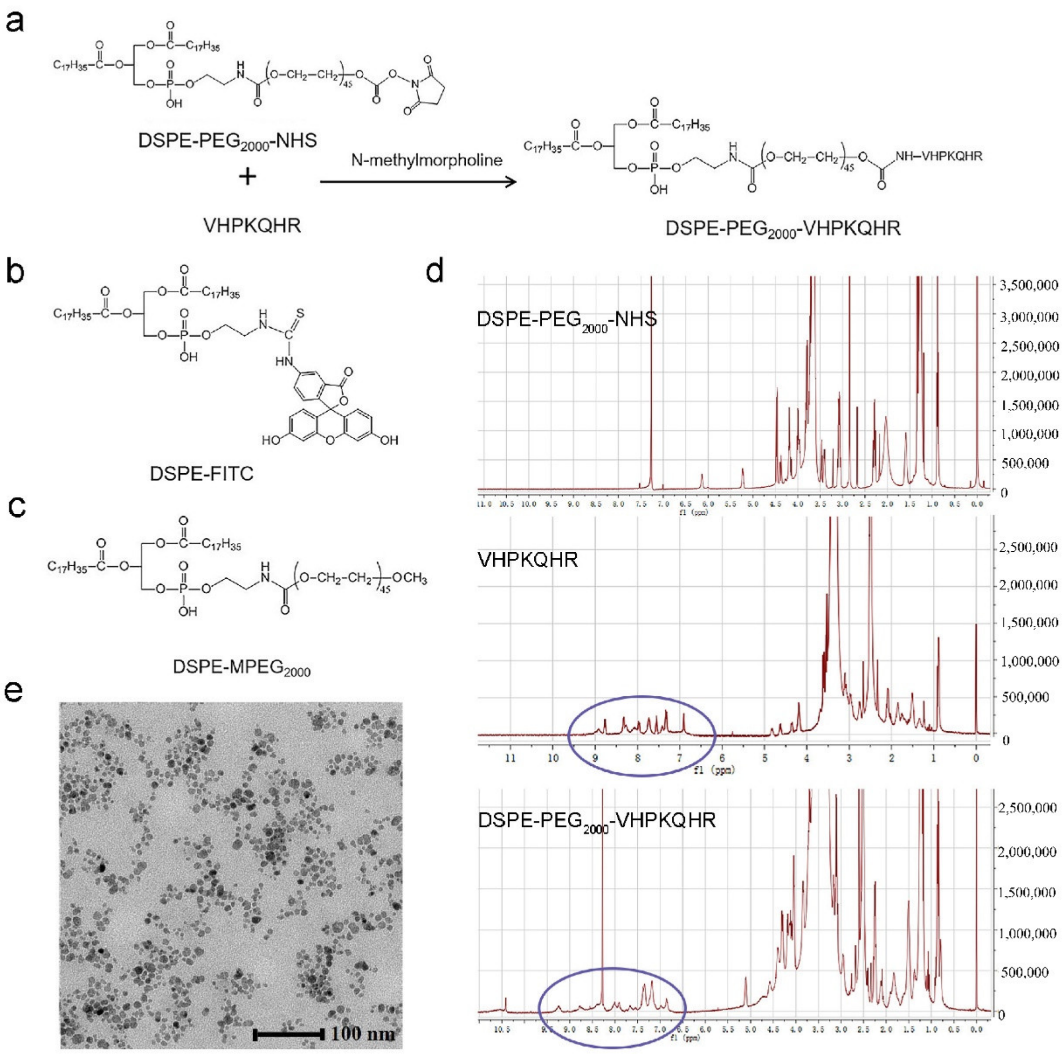

Supplementary Figure S1. Structure and characterization of materials used in the synthesis of Rap/Fe_3O_4@VHP-Lipo. (a) Synthetic chemical reaction formula of DSPE-PEG_{2000}-VHPKQHR. (b) The chemical structure of DSPE-FITC. (c) The chemical structure of DSPE-$MPEG_{2000}$. (d) ^1H NMR of reaction substrate DSPE-PEG_{2000}-NHS, VHPKQHR peptide and reaction product DSPE-PEG_{2000}-VHPKQHR. (e) The morphology of USPIO-BSA (Fe_3O_4) under TEM.

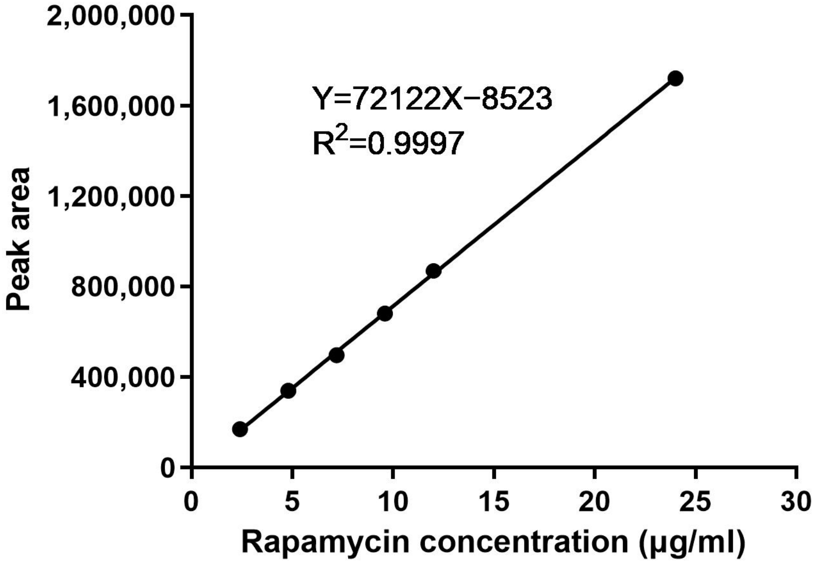

Supplementary Figure S2. Establishment of a method for the analysis of rapamycin by liquid chromatography. Linear relationship between rapamycin concentration and peak area measured by HPLC.

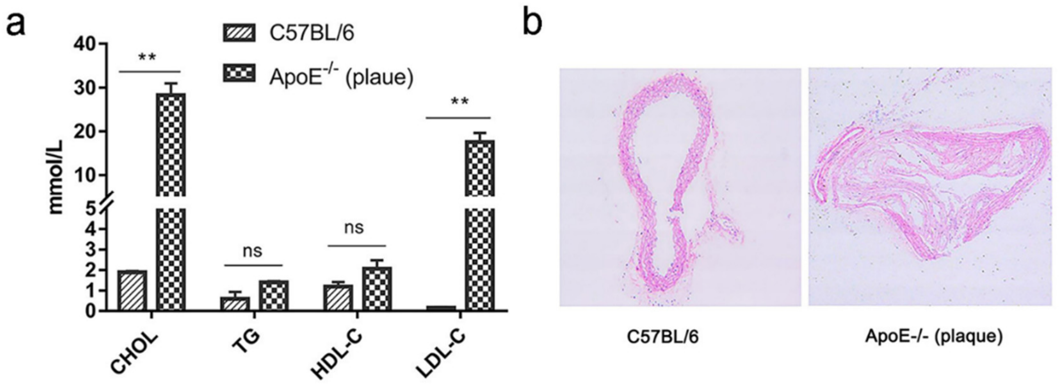

Supplementary Figure S3. Construction of atherosclerosis model mouse. (a) Comparison of blood lipid indexes of C57BL/6 mice fed with normal diet and ApoE$^{-/-}$ mice fed with high-fat diet (HFD) for 4 months. (b) H&E stained sections of isolated aorta of C57BL/6 mice and ApoE$^{-/-}$ mice with plaque. (** $p < 0.01$, ns $p > 0.05$, mean = ±SD).

a

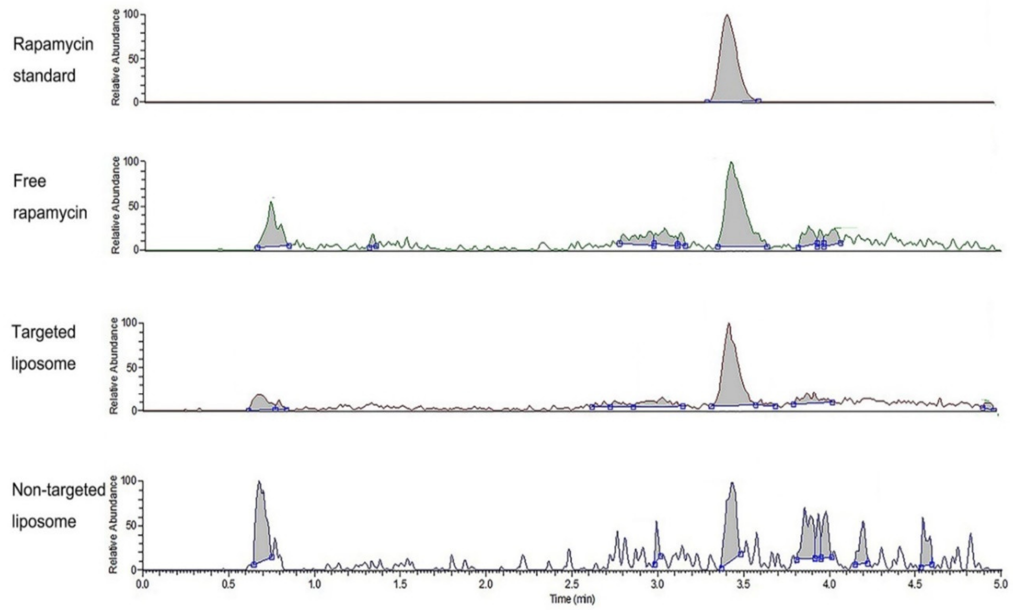

b

Compound Optimization Results						
Compound Name	Precursor m/z	Product m/z	Collision Energy (V)	RF Lens (V)	Intensity	Source Fragmentation (V)
RAPA	936.7	607.333	46.809	248.764	76509.962	0
RAPA	936.7	409.236	52.371	248.764	319640.833	0
RAPA	936.7	453.222	49.034	248.764	108796.497	0

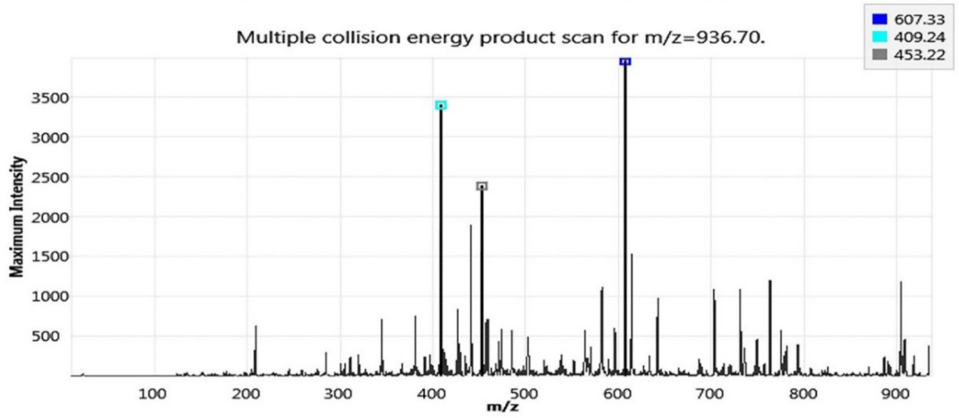

Supplementary Figure S4. Exploring the Chromatographic and Mass Spectrometric Conditions of Rapamycin. (a) The retention time of rapamycin in the liquid chromatography column of rapamycin standard, free rapamycin treated group blood samples, Rap/Fe_3O_4@Lipo treated group blood samples and Rap/Fe_3O_4@VHP-Lipo treated group blood samples. (b) The mass spectrum of rapamycin and the three most significant fragment ions.

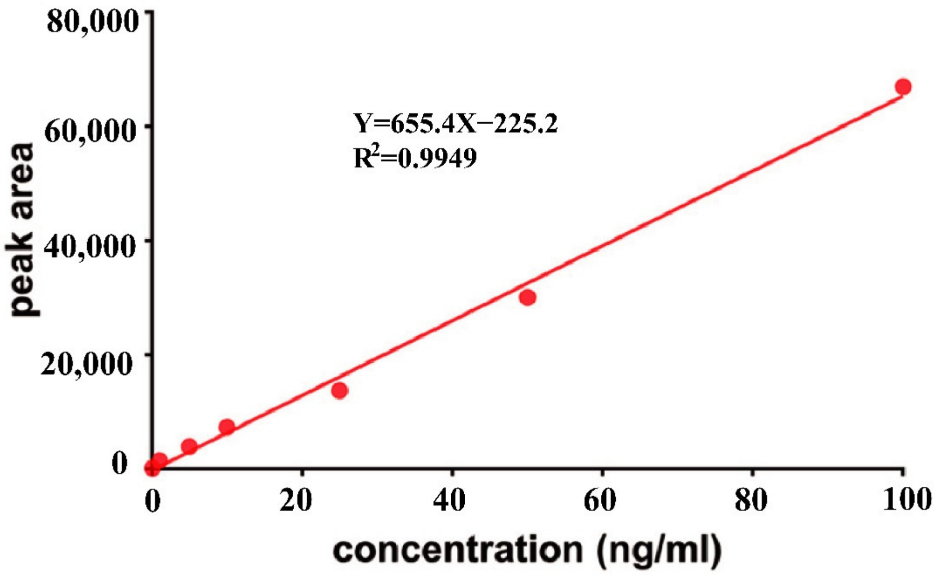

Supplementary Figure S5. Establishment of a liquid mass spectrometer method for the analysis of rapamycin concentration. Linearity between rapamycin concentration and peak area determined by LC-MS.

2 Supplementary table

Supplementary Table S1. Determination of the drug concentration in liposomes by HPLC. Take 200 μL of liposome solution, dilute to 10 mL with methanol and measure drug concentration by HPLC.

Area of peak	Determination of concentration	Drug loading	Encapsulated	The content of rapamycin
1327176.6	18.52μg/ml	5.5%	92.6%	0.926 mg/ml

Theoretical concentration =1000μg/ml *0.2/10=20 μg/ml, Encapsulated =18.52/20*100%=92.6%
The content of rapamycin =1 mg/ml*92.6%=0.926 mg/ml
Drug loading =0.926*2/(0.926*2+30+2)*100%=5.5%
